# Profiles of Cognitive-Motor Interference During Walking in Children: Does the Motor or the Cognitive Task Matter?

**DOI:** 10.3389/fpsyg.2018.00947

**Published:** 2018-06-13

**Authors:** Nadja Schott, Thomas J. Klotzbier

**Affiliations:** Department of Sport and Exercise Science, University of Stuttgart, Stuttgart, Germany

**Keywords:** children, locomotion, dual task, Trail-Walking-Test, visuo-spatial working memory, executive attention network, cognitive-motor interference

## Abstract

The evidence supporting the effects of age on the ability to coordinate a motor and a cognitive task show inconsistent results in children and adolescents, where the Dual-Task Effects (DTE) – if computed at all – range from either being lower or comparable or higher in younger children than in older children, adolescents and adults. A feasible reason for the variability in such findings is the wide range of cognitive tasks (and to some extend of motor tasks) used to study Cognitive-Motor Interference (CMI). Our study aims at determining the differences in CMI when performing cognitive tasks targeting different cognitive functions at varying walking pathways. 69 children and adolescents (boys, *n* = 45; girls, *n* = 24; mean age, 11.5 ± 1.50 years) completed higher-level executive function tasks (2-Back, Serial Subtraction, Auditory Stroop, Clock Task, TMT-B) in comparison to non-executive distracter tasks [Motor Response Task (MRT), TMT-A] to assess relative effects on gait during straight vs. repeated Change of Direction (COD) walking. DT during COD walking was assessed using the Trail-Walking-Test (TWT). The motor and cognitive DTE were calculated for each task. There were significant differences between 5th and 8th graders on single gait speed on the straight (*p* = 0.016) and the COD pathway (*p* = 0.023), but not on any of the DT conditions. The calculation of DTEs revealed that motor DTEs were lowest for the MRT and highest for the TWT in the numbers/letters condition (*p* < 0.05 for all comparisons). In contrast, there were cognitive benefits for the higher-order cognitive tasks on the straight pathways, but cognitive costs for both DT conditions on the COD pathway (*p* < 0.01 for all comparisons). Our findings demonstrate that DT changes in walking when completing a secondary task that involve higher-level cognition are attributable to more than low-level divided attention or motor response processes. These results specifically show the direct competition for higher-level executive function resources important for walking, and are in agreement with previous studies supporting the cognitive-motor link in relation to gait in children. This might be in line with the idea that younger children may not have adequate cognitive resources.

## Introduction

Can I have your attention, please? Multitasking is already embedded in the daily lives of children and adolescents: 70% of high-school students spent half of their time in class with recreational activities and other non-academic related activities while attending lectures (Lauricella and Kay, 2010); however, studies show that using a laptop or a cell phone during class limit recall of class material (Hembrooke and Gay, 2003). Adolescents spend 60% of the time they set aside for homework switching between homework and other activities (e.g., emails, instant messaging; playing video games; navigating through their home while carrying a tray of food; Foehr, 2006). Recently, Baumgartner and Sumter (2017) showed that children aged 6–13 years find it difficult to focus their attention on a main activity in the presence of appealing media distractors, e.g., walking, crossing a street while using a mobile was found to be the primary explanation for increasing rates of pedestrian injuries (Byington and Schwebel, 2013; Thompson et al., 2013; Retting and Rothenberg, 2015). Also, most other tasks of everyday life (e.g., crossing a room while carrying an object; driving a car while making a call) or in sport settings (e.g., dribbling a ball while scanning around for a teammate to whom to pass) are not done in isolation, requiring the individual to perform two or more tasks either simultaneously or in rapid succession.

The ability to complete this type of tasks without errors requires management of attention and task prioritization so that all tasks may be completed efficiently, but attentional resources are not infinite (Pashler, 1994). Coordinating a motor and a cognitive task (dual-tasking, DT) might result in performance decrements in one or both tasks, relative to performance of each task separately. This occurs when the two tasks interfere with one another, known as cognitive-motor interference (CMI), and is thought to be a proof of capacity limitation in cognitive abilities (Watanabe and Funahashi, 2014). An increase in CMI during gait has been shown in younger and older adults with and without multiple clinical conditions such as concussion, Multiple Sclerosis, Parkinson, or dementia resulting in impaired functional gait performance and increased risk of falls (Wajda and Sosnoff, 2015; Smith et al., 2016; Belghali et al., 2017; Klotzbier and Schott, 2017; Schott, 2017; Fino et al., 2018). However, data is limited for both healthy children and adolescents.

The few studies that have examined cognitive-motor interference in typically developing children and adolescents have primarily used walking straight ahead as their motor task (Whitall, 1991; Huang et al., 2003; Cherng et al., 2007; Schaefer et al., 2010, 2015; Anderson et al., 2011; Krampe et al., 2011; Boonyong et al., 2012; Beurskens et al., 2015, 2016; Hagmann-von Arx et al., 2016; Hinton and Vallis, 2016; Chauvel et al., 2017). Walking is thought to be an automated skill in adulthood, successfully coordinated with only minimal use of attention-demanding executive control resources (Clark, 2015; Bisi and Stagni, 2016). Kraan et al. (2017) describe the changes of gait across childhood as steady and similar to adults around 7–8 years of age with changes in gait speed from 0.6 to 1.1 m/s. However, they also point out that temporal and spatial parameters will improve with subtler changes around 11–15 years. Hausdorff et al. (1999) argue that the development of the central nervous system has a greater impact on gait variability than anthropomorphic characteristics. This is even more evident, when examining complex situations such as navigating around or over obstacles (e.g., avoiding other individuals on a sidewalk or during sports events; walking through narrow openings). To maintain balance with these challenging aspects, individuals need to constantly modify their movement patterns to propel in response to environmental constraints using reactive or anticipatory strategies. This is referred to as adaptive locomotion (Higuchi, 2013). Children use different anticipatory strategies than adults, making last minute adjustments, while adults plan well ahead of upcoming obstacles. For instance, Vallis and McFadyen (2005) found in middle-aged children (9–12 years of age) reductions in gait speed and step length only two steps and one step prior to obstacle circumvention, respectively, while adults maintain a constant speed and step length. Therefore, the automaticity of the locomotor system depends also heavily on gait task difficulty (Schott et al., 2016).

In recent years, researchers focus especially on the relationship between DT performance, the attention network, and executive functions (e.g., planning, shifting, inhibition, dividing of attention) to shed light on higher-order cognitive functions (McFadyen et al., 2015; Walshe et al., 2015). Executive functions continue to develop throughout childhood with increasing efficiency at around 7 years of age (Davidson et al., 2006) well into adolescence (Pozuelos et al., 2014) and early adulthood (Anderson et al., 2011). However, the rate of this change is driven by both age and performance changes over time, e.g., a recent study demonstrated that performance in a task similar to the Wisconsin Card Sorting Task increased rapidly in childhood and early adolescence (8–14 years) after which it stabilized (Koolschijn et al., 2011). In another recent study, Boelema et al. (2014) revealed developmental trajectories for the maturation of different executive function tasks during adolescence: While inhibition reached mature levels first, information processing speed, working memory, and shift attention exhibited largest change rates and therefore most maturation between the transitions from childhood to adulthood (Schott and Klotzbier, 2018). In spite of this, evidence for the maturational timeline of executive function during childhood and adolescence is inconsistent, with findings of the rate of improvement varying considerably between individuals (Shanmugan and Satterhwaite, 2016). As Yang et al. (2017) point out, it remains unknown how these cognitive functions interact when subjects need to solve two tasks at the same time. Moreover, the refinement of cognition and DT ability results from the emergence of networks of coordinated activity spanning multiple distributed regions (Petersen and Sporns, 2015). Due to these changes one can assume that DT performance should also go through significant change during this period (Yang et al., 2017). However, to date only few studies have investigated DT in typically developing children, who exhibiting typically greater vulnerability to Dual Task Effects (DTE). The evidence supporting the effects of age on the ability to coordinate a motor and a cognitive task show inconsistent results in children and adolescents, where the DTE – if computed at all – range from either being lower or comparable or higher in younger children (4–6 years) than in older children (7–12 years), adolescents and adults (Whitall, 1991; Huang et al., 2003; Cherng et al., 2007; Schaefer et al., 2010, 2015; Anderson et al., 2011; Krampe et al., 2011; Boonyong et al., 2012; Beurskens et al., 2015, 2016; Hagmann-von Arx et al., 2016; Hinton and Vallis, 2016; Chauvel et al., 2017; see also Saxena et al., 2017 for an excellent review). For instance, Boonyong et al. (2012) found that children aged 5 and 6 years, and 7–16 years, use a more careful strategy (e.g., reduced gait speed and step length), than that of adults during obstacle crossing while performing an Auditory Stroop-Task. Hagmann-von Arx et al. (2016) reported that gait performance in children and adolescents (6.7–13.2 years), was stronger affected in a motor dual-task condition in which children were asked to fasten and unfasten a shirt button than in a cognitive dual-task condition in which children were asked to listen to and memorize digits while walking. However, no age-dependent dual-task effects on gait velocity in the cognitive dual-task condition were found. Moreover, Schaefer et al. (2010) comparing 9-year old children and adults while performing an 1- to 4-Back task during treadmill walking showed an increase in variability of spatio-temporal parameters, but improved cognitive performance under low cognitive loads. Yang et al. (2017) suggest to include not only children aged 10 years of age and less, but due to the continuous development of cognitive functions underlying DT ability (e.g., time monitoring, prospective memory, planning) children older than 10 years may provide new insights into DT development and its underlying processes during late childhood.

These findings may be interpreted from a methodological perspective and/or from the perspective of the cross-domain competition model (Lacour et al., 2008), which postulates that a motor and a cognitive task compete for attentional resources. Its main prediction is that maintaining kinematic gait parameters should be less efficient in DT than ST conditions, which in turn depends on the complexity of the selected task as well as the development of the selected cognitive domain. Recent reviews by Ruffieux et al. (2015) and Saxena et al. (2017) examining balance and walking performance under dual-task conditions discuss several methodological issues in existing studies at length. Saxena et al. (2017) suggest seven criterions to improve overall quality of studies: appropriateness of single tasks (ST), equation of tasks, calculation of DTEs, DTE for each ST, randomization of task order, practice effects, and clear instructions. Both reviews conclude, that single-task performance of the secondary task is not assessed, thus the calculation of DTEs is not permitted. Additionally, the wide range of cognitive tasks [and to some extend of motor tasks (mostly walking straight ahead; balance on two feet or one foot)] is another possible reason for the variability in findings used to study CMI with different types of cognitive and motor DT leading to different types of cognitive-motor interference (Kraan et al., 2017). To date, the cognitive tasks used in CMI studies include auditory, verbal and visuo-spatial working memory (Digit Recall; N-Back, Digit Span), inhibition (Stroop), and verbal fluency. As pointed out earlier, different components of EF have been shown to develop at different rates; therefore, the results for the relationship of motor and cognitive performance rely highly on the selected cognitive task. Most studies dealing with CMI use only one cognitive task. Researchers using different motor and multiple cognitive tasks have failed to refer to the cognitive functions targeted by the secondary tasks or did not compare the changes in CMI caused by two different tasks (Patel et al., 2014). However, different cognitive tasks may interfere with walking to a different extent, depending on the cognitive demands of the tasks (Schott et al., 2016).

The purpose of this project was to determine the differences in children’s and adolescents CMI when performing cognitive tasks targeting different cognitive functions at varying walking pathways. Thus, the aims of this study were (1) to examine the effect of higher-level executive function tasks (2-Back, Serial Subtraction, Stroop- and Clock-task) in comparison to non-executive distracter tasks (motor task) on motor and cognitive costs of dual-task walking and (2) to determine the effect of straight versus Change-of-Direction (COD) -walking on motor and cognitive costs of dual-task walking.

We hypothesized that higher motor cost will be associated with a cognitive task which demands higher-level compared to lower-level executive processes, and that these costs will be higher in younger compared to older children. Higher motor and/or cognitive costs would indicate the requirement of greater attentional resources for that cognitive task, under DT conditions. We further hypothesized that compared to walking on a straight pathway COD walking while dual-tasking would decrease the performance on the cognitive tasks, i.e., increase the cognitive cost of dual-task walking.

## Materials and Methods

### Participants

Sixty-nine fifth-, and eighth-graders voluntarily participated in the study (boys, *n* = 45; girls, *n* = 24; mean age, 11.5 ± 1.50 years, range 10–14 years). As mature walking patterns with upper body stability, similar to adults occur at 7–10 years of age in healthy children (Mazzà et al., 2010), we chose an age range of 10 years or older. All children and adolescents were recruited from the same mainstream school (middle socioeconomic class) in the south of Germany. They were right handed and right footed except for seven children who were left handed and left footed. None of these children had known visual, neurological or motor deficits (based on parent’s report).

Local ethics committee approved the study procedures, designed in accordance with the Declaration of Helsinki on ethical standards, legal requirements and international norms. Written informed parental consent was obtained for each child and all children assented to participate.

### Experimental Protocol

Each child performed both single and dual-tasks with low and high task complexity (see **Table 1**). They completed 5 single-task walking trials on a straight pathway (walking without a cognitive demand), 3 single-task walking trials on a COD pathway, 5 dual-task walking trials on a straight pathway [walking while completing (a) Auditory Stroop, (b) N-Back, (c) Serial Subtraction, (d) Clock and (e) Auditory Motor Task], and 2 dual-task walking trials on a COD pathway (walking while concurrently completing a cognitive test; TWT-2 and TWT-3). During all trials on a straight pathway, participants walked for 60 s at a self-selected pace around a 5 m × 5 m rectangle. All trials on the COD pathways (Schott, 2015) had a length of 41 m in total.

**Table 1:** Single task (ST) and dual task (DT) conditions by task complexity (created after McIsaac et al., 2015).

Type of tasks	Task complexity	
	Low	High
	
Single motor	Walking on a straight pathway	Walking on a COD pathway
Single cognitive	• Trail-Making-Test A • Auditory Motor Task (AMT)	• Trail-Making-Test B • Auditory Stroop Task • Auditory 2-Back • Serial Subtraction Task • Clock Task
Dual motor-cognitive	• Walking on a straight pathway while responding to an auditory signal (AMT) • Walking on a COD pathway while stepping on numbered targets in a sequential order (TWT-2)	• Walking on a straight pathway while completing the (a) Auditory Stroop, (b) N-Back, (c) Serial Subtraction, and (d) Clock Task • Walking on a COD pathway while stepping on targets with increasing sequential numbers and letters (TWT-3)

*TWT, Trail-Walking-Test; COD, change of direction.*

Participants were also asked to perform all cognitive tasks while seated approximately 60 cm from a 17″ blank screen laptop. They first received standardized instructions on how to perform the cognitive task. After that a familiarization procedure was carried out. E-Prime stimulus software (Psychology Software Tools, Pittsburgh, PA, United States) presented the stimuli and collected responses (accuracy, RT). Each task (single and dual) ran for 60 s in both conditions, with accuracy and/or reaction times recorded. Only the Serial Substraction Task (SST), and the Trail-Making-Test (TMT) were manually recorded.

During the dual motor-cognitive conditions, children and adolescents carried a wireless mouse in their right hand for tasks that required a button-press response. No explicit task prioritization was offered. Participants were instructed to walk as in the ST walking condition while achieving the fastest and fully accurate responses on the cognitive tasks as in the seating-only condition.

Motor task performance was either measured as the distance walked in 60 s in each walking condition (straight pathway) or as duration (COD pathway): gait speed was then calculated for each participant using distance in meters and time in seconds. It was obtained by dividing the distance traveled by the time to cover that distance. Cognitive task performance was either measured as the number of correct responses in 1 min in each condition (straight pathway) or as duration (COD pathway).

For each participant, both single-task and dual-task conditions were randomized within each condition for each participant. All single-task conditions preceded dual-task conditions to maintain consistency. To ensure that each participant understood each task they got practice trials running for 30 s. The experiment took about 90 min in total for each child.

### Measures

#### Demographic Information, Subjective Motor Performance and Physical Activity

Basic demographic information as well as medical history were acquired by interviewing the children and their parents. Height and weight of the participants were measured, and the body mass index (BMI, kg/m^2^) was calculated. Since body composition is an important independent contributor to motor and cognitive performance (Davis et al., 2015). We categorized BMI^1^ according to international age- and gender-specific reference values.

The MABC-2 Checklist (Henderson et al., 2007) was created to evaluate children’s motor behavior in different everyday situations of life, such as in the classroom, recreational and physical education activities and in everyday situations of personal care (Sections A, B) and in non-motor factors (Section C) that might affect movement, e.g., lack of confidence or impulsiveness. It is designed to identify children with motor difficulties in the age range 5–12 years. The Total Motor Score (TMS) is the sum of the 30-item scores (Sections A+B), the higher the TMS, the poorer the performance. The Coefficient Alpha of both groups in this study was 0.93 for all 43 items (together), 0.81 for section A (static/predictable), 0.81 for section B (dynamic/unpredictable), and 0.63 for section C (non-motor factors).

Furthermore, children were asked in which organized activities (participation through a formal club, maximum three different activities) they had participated over the past 12 months (see also Schott et al., 2016). Next, children were asked how many days a week, and minutes per session, they had participated in that particular activity. Total physical activity (h/week) was calculated as follows: (frequency 1 × duration 1) + (frequency 2 × duration 2) + (frequency 3 × duration 3). Additionally, children were asked, “Over the past 7 days, on how many days were you physically active for a total of 60 min or more per day?”

#### Cognitive Tasks – Straight Pathway

We adopted four cognitive tasks from Walshe et al. (2015), which are generally used to evaluate DT performance (lower-level decision-making: Auditory Motor task; higher-level executive process: Clock Task, N-Back, Serial Subtraction). Additionally, we used an Auditory Stroop Task. The duration of each task was 60 s.

The *Auditory Motor Task (AMT)* is a simple reaction time task and evaluates the processing speed of the central nervous system as well as the coordination between the sensory and the motor system. In this task, participants were presented a single auditory tone (16-Bit WAV file; 705 kbit; 1000 ms long with a 3000 ms response window from start of stimulus) at randomly varied delay intervals, (500 ms, or 1000 ms). They were instructed to quickly respond with a mouse click.

The *Clock Task* (Haggard et al., 2000) is a visuo-spatial working memory task, which requires participants to respond to an auditory speech sample announcing a time, e.g., one-twenty-five (female voice; 16-Bit WAV file; 1411 kBit; 1000 ms long with a 3000 ms response window from start of stimulus; 500 ms stimulus interval). Dividing the clock in a left and a right half participants determined whether the two hands of the clock at the given time were in the same half (left mouse click) or opposite halves (right mouse click).

An *Auditory 2-Back Task* (Owen et al., 2005) was used to assess working memory. In this study, participants heard a sequence of numbers (e.g., “3–8–3”) from a female voice (Toronto Noun Pool; 16-Bit WAV file; 1536 kbit/s; 1500 ms window with randomly varied delay intervals between 2000 and 2500 ms), presented one at a time, and were required to respond with a button press (wireless mouse) to the relevant auditory numerical stimuli and to withhold responses to distractor stimuli. The stimulus sequence was different for ST and DT conditions to control for learning effects.

The *Serial Subtraction Task (SST)* measures attention, mental calculation and working memory (Karzmark, 2000). Participants were required to count backward in threes (e.g., “100–97–94”) as quickly and as accurately as possible. During a practice phase, the participants counted backward from 25 in threes.

The *Auditory Stroop Task (AST)* is a modification of Stroop and is used to study cognitive control and conflict monitoring (Morgan and Brandt, 1989). The participants responded manually via wireless mouse clicks to tonality, but not to the words: they heard the words “high” and “low” spoken in either a high pitch (360 Hz) or a low pitch (180 Hz) voice. The participants were instructed to indicate the pitch of the word they heard (ignoring the actual word presented) by responding “high” (left click) or “low” (right click) as accurately and as quickly as possible.

#### Cognitive Tasks – COD – Pathway

The *Trail-Making-Test* (TMT, Reitan, 1955) was used to examine executive function under fine motor control conditions. Originally, the paper-and-pencil test consists of two parts: During Part A (TMT-A; attention, visual scanning, motor speed and coordination), subjects are instructed to connect encircled numbers (1–25) randomly distributed on a white sheet of paper. In Part B (TMT-B; mental flexibility and working memory in addition to the abilities assessed by part A), participants are asked to connect randomly positioned numbers (1–13) and letters (A–L) in an ascending number-letter sequence (1–A–2–B– etc.). Additionally, we included a motor speed condition (TMT motor speed) as the ST condition: participants trace over a dotted line connecting circles on the page (trail of the same length compared to TMT A and B), in order to test their ability to adapt movement accuracy to spatial constraints based on incoming visual feedback with temporal pressure (Klotzbier and Schott, 2017). During performance, errors were immediately corrected by the examiner instructing the participant to go back to the last correct item, thus increasing the time taken to complete the test. The trials were timed using a stopwatch to the nearest 0.01 s; also, the number of errors was recorded. Due to the longer total trail length of TMT B compared to TMT A (Gaudino et al., 1995) and TMT motor speed we report the speed (cm/s) instead of the total duration.

The *Trail-Walking-Test* (TWT, Schott, 2015) was used to examine executive function under gross motor control conditions. Cones with flags are placed randomly at each of 15 positions in a 16-m^2^ area (4 × 4 m). 30-cm diameter circles were drawn around each cone. The participants were required to (1) follow a line of connecting circles (TWT-1, ST), (2) step on numbered targets in a sequential order (i.e., 1–2–3; TWT-2, DT), and (3) step on targets with increasing sequential numbers and letters (i.e., 1–A–2–B–3–C; TWT-3; DT). In addition, participants were instructed to move from one flag to the next in an ascending order as quickly, but as accurately as possible. However, no priority was given to one domain or the other. Trials were considered successful when the participant did not knock over a cone, step on the circle (motor errors), and did not walk in the wrong direction (sequencing and shifting errors; Klusman et al., 1989) or exhibit extended search patterns. During performance, sequencing and shifting errors were immediately corrected by the examiner instructing the participant to go back to the last correct item; motor errors were only recorded. The trials were timed using a stopwatch to the nearest 0.01 s following a standard procedure. Gait speed was calculated for each participant using distance in meters and time in seconds. It was obtained by dividing the distance traveled 41 m by the time to cover that distance. Each condition was performed three times.

### Data Analysis

Statistical analyses were implemented on SPSS v.24 (SPSS, Chicago, IL, United States). We first explored dependent variables to examine missing data points, normality of distributions (tested by Kolmogorov–Smirnov tests), and presence of outliers (defined by the Explore command of SPSS v.24). An alpha level of 0.05 was used for all statistical tests. Potential baseline group differences for continuous variables (i.e., age, height, weight, BMI, physical activity, VO_2_max) were assessed using ANOVAs, and categorical demographic variables (i.e., gender, weight category) were compared by chi-square test.

For measuring the performance level in the cognitive task, the computation of the “correct cognitive response” (CCR) was adopted from MacLean et al. (2017). The CCR score in the ST conditions was calculated by dividing the number of correct responses by the time taken (60 s) to produce a response rate per second. This result was then multiplied by the ratio of correct responses to total responses, to take error into account, with higher CCR scores indicating better cognitive performance. The CCR scores in the DT conditions were first calculated as the number of correct responses given in the DT, divided by the time taken for each individual DT condition and this result was then multiplied by the ratio of correct responses to total responses, to adjust for errors.

To quantify the effect of dual tasking on both motor and cognitive parameters we compared the absolute values for all cognitive and motor parameters between single- and DT-conditions. To compare the motor and cognitive function across the different DT conditions, the motor and cognitive DTEs calculated according to the common formula (Plummer and Eskes, 2015):

(1) $$\begin{array}{c} \mathrm{DTE}(\%)=\frac{\left(\mathrm{Dual}task-Single\mathrm{task}\right)}{\mathrm{Single}\mathrm{task}\mathrm{time}}*100 \end{array}$$

Negative DTE values indicate that performance deteriorated in the DT relative to the ST (i.e., DT cost), whereas positive DTE values indicate a relative improvement in performance in the dual-task (i.e., DT benefit) (Plummer and Eskes, 2015, p. 3). It is important to examine change in both activities, because motor performance can decline in one or both of the activities performed simultaneously when they exceed the available attentional resources; thus, we examined motor and cognitive DTEs.

Correlation analysis between motor and cognitive performance and age, sex, exercise, subjective motor performance, and Vo_2_max was performed using Pearson’s correlation (*r*) or Spearman’s rank correlation (*rSp*) in cases of not normally distributed variables. In a regression analysis, we included most relevant confounders (*|r/rSp*| > 0.2) that may interact with DT gait performance. Due to the high number of regressions performed, the level of significance was set to *p* < 0.01 to reduce the probability of alpha error accumulation.

To analyze the effect of the different task conditions on gait speed, each variable was analyzed using a 2 × 6(3) (six different conditions on the straight pathway; three different conditions on the COD pathway) repeated measure analysis of variance (ANOVA) with task conditions as the within-group factor and age group as between factors. Paired *t*-tests were performed between cognitive performance scores in the sitting and walking conditions for each cognitive task. The motor and cognitive costs across the 5 (straight pathway)/2(COD pathway) DT conditions were compared using a 2 × 5(2) repeated measures ANOVA. Significant findings were followed up with *post hoc* analysis to determine the effect of specific cognitive tasks on gait speed (motor function).

Effect size for all ANOVAs was reported using partial eta squared ($\eta_{p}^{2}$), with a small effect defined as 0.01, a medium effect as 0.06, and a large effect as 0.14 (Cohen, 1988). Repeated measures sphericity issues were addressed with the Greenhouse Geisser correction. When ANOVAs were statistically significant, *post hoc* comparisons were performed using the Bonferroni correction. The level of significance for *post hoc* comparisons was set at 0.05.

## Results

### Participants

**Table 2** depicts the demographic, subjective motor performance, physical activity, and general cognitive performance measures. Group comparisons showed that children (29 boys, 13 girls) and adolescents (16 boys, 11 girls) differed on exercise duration, subjective motor performance (TMS; total motor score of the MABC-2 Checklist A and B), and Vo_2_max with adolescents outperforming children. Furthermore, girls (46.2 ± 3.59) exhibited lower Vo_2_max scores compared to boys (52.0 ± 4.08), *t*(67) = -5.85, *p* < 0.001, *d* = 1.07.

**Table 2:** Demographics, exercise, motor performance, and physical fitness of children by grade (means ± standard deviation).

	5th grade	8th grade	Statistical analysis – *p*-value
	(*n* = 42)	(*n* = 27)	
Age (years)	10.3 ± 0.53	13.2 ± 0.24	*t*(67) = -24.0, *p* < 0.001, *d* = 0.70
Sex (boys/girls)	29/13	16/11	*CHI^2^*(1) = 0.69, *p* = 0.405
BMI (kg/m^2^)	16.4 ± 1.90	18.8 ± 1.34	*t*(59) = -5.59, *p* < 0.001, *d* = 0.25
Exercise (min/wk)	177 ± 94.6	290 ± 165	*t*(77) = -3.22, *p* = 0.003, *d* = 0.17
MABC-2 checklist			
A and B (0–90)	4.95 ± 6.25	2.26 ± 3.21	*t*(64) = 2.35, *p* = 0.022, *d* = 0.54
C (0–13)	2.17 ± 2.19	1.56 ± 2.21	*t*(67) = 1.13, *p* = 0.263, *d* = 0.28
PACER (laps)	42.5 ± 14.4	63.1 ± 19.6	*t*(67) = -5.02, *p* < 0.001, *d* = 1.20
Vo_2_max	49.2 ± 5.10	51.3 ± 4.00	*t*(67) = -1.83, *p* = 0.071, *d* = 0.06

*BMI, Body-Mass-Index; MABC-2 Movement Assessment Battery.*

### Influence of Age, Sex, Exercise, Subjective Motor Performance, and Vo_2_max on Motor and Cognitive Performance

Supplementary Table S1 shows univariate correlations between dependent and independent variables. Overall, we found only small to moderate correlations between age, sex, BMI, physical activity, subjective motor performance, Vo_2_max and the different dual tasks (DT). The results of regression analysis for motor and cognitive performance, motor and cognitive DTEs are summarized in Supplementary Table S2. Only age showed a significant relationship to DT performance in almost all tasks on the straight pathway.

### Group Differences on Gait Speed and Cognitive Performance in ST- and DT-Conditions

#### Gait Performance

**FIGURE 1:**
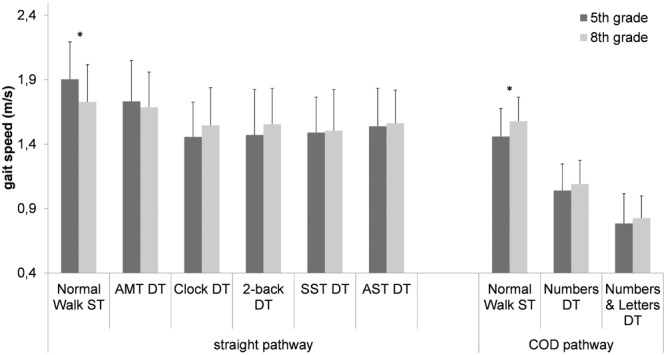
Gait speed (means ± standard deviation) on a straight and a COD pathway as a function of type of cognitive task and age group (5th grade, 8th grade). ^∗^ age groups significantly different from each other (ST, single task; DT, dual task; AMT, Auditory Motor Task; SST, Serial Subtraction Task; AST, Auditory Stroop Task).

There were significant differences between 5th and 8th graders on single gait speed on the straight pathway and the COD pathway, but not on any of the DT conditions (see **Figure 1** and Supplementary Table S3). Lower- and higher-level cognitive tasks had a significant effect on gait speed with a significantly lower gait speed during all dual-task conditions compared to the single-task walking on a straight pathway, [*F*(4.2,276) = 40.4, *p* < 0.001, $\eta_{p}^{2}$ = 0.376] as well as on the COD-pathways [*F*(1.8,122) = 343, *p* < 0.001, $\eta_{p}^{2}$ = 0.836]. However, there were no significant differences in gait speed between the Clock Task, 2-Back, SST, and Stroop, but significant differences between the TWT-1, TWT-2, and the TWT-3 condition (*p* < 0.001). Higher task complexity resulted in a higher magnitude of decline in gait speed.

#### Cognitive Performance

**FIGURE 2:**
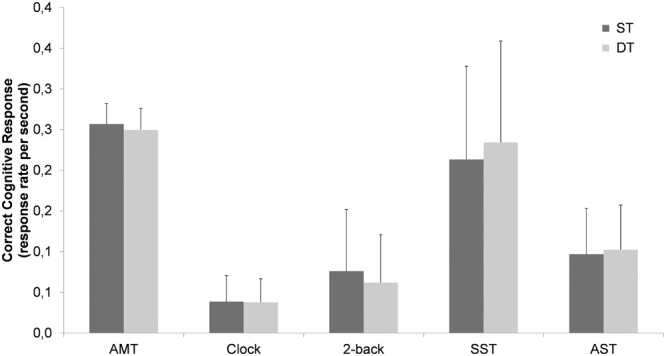
Cognitive performance (Correct Cognitive Response, CCR) (means ± standard deviation) by walking condition (ST, single task; DT, dual task; AMT, Auditory Motor Task; SST, Serial Subtraction Task; AST, Auditory Stroop Task).

A significant main effect for condition (CCR-rate) was found, [*F*(1.76,108) = 187, *p* < 0.001, $\eta_{p}^{2}$ = 0.754] indicating better performance on the Auditory Motor Task and the Serial Subtraction Task compared to the Auditory Stroop Task, 2-Back Task, and the Clock Task. *Post hoc* tests conform that all cognitive tasks differ significantly from each other. Furthermore, the interaction for ST vs. DT by grade [*F*(1,61) = 3.79, *p* = 0.056, $\eta_{p}^{2}$ = 0.058] approached significance as well as the interaction between condition and ST vs. DT, [*F*(2.11,129) = 2.55, *p* = 0.079, $\eta_{p}^{2}$ = 0.040]. Overall, children (5th graders) performed better in the DT conditions compared to the ST conditions (CCR: 0.139 vs. 0.130), while the adolescents (8th graders) performed better in the ST conditions compared to the DT conditions (CCR: 0.146 vs. 0.136). The CCR was decreased under DT conditions only for the Auditory Motor Task, the 2-Back Task, and the Clock Task, but increased for the Serial Subtraction Task and the Auditory Stroop Task (see **Figure 2**).

ANOVAs with repeated measures indicated that there were significant differences between all three conditions for the Trail-Making-Test [*F*(1.35,90.2) = 223, *p* < 0.001, $\eta_{p}^{2}$ = 0.769] as well as the Trail-Walking-Test [*F*(1.82,122) = 343, *p* < 0.001, $\eta_{p}^{2}$ = 0.836] with lower speeds for the tasks with higher task difficulty (see also Supplementary Table S3). There were no significant interactions for age group × task.

#### Motor and Cognitive Dual Task Effects

**FIGURE 3:**
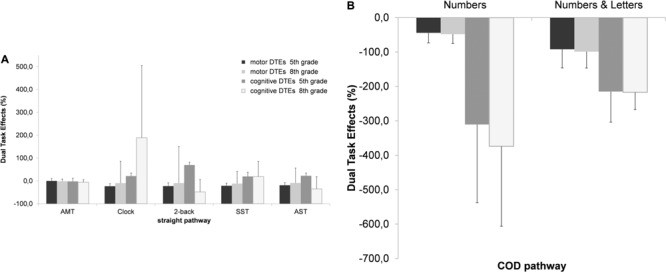
Motor and cognitive DTEs (%, means ± standard deviation) on **(A)** a straight and **(B)** a COD pathway as a function of type of cognitive task and age group (5th grade, 8th grade) (%) (DTE, dual task effects; AMT, Auditory Motor Task; SST, Serial Subtraction Task; AST, Auditory Stroop Task) (standard deviations can be found in the Supplementary Material).

A comparison of motor DTEs for the straight pathway revealed that motor cost was significantly lower in the simple motor response condition compared to all other conditions [*F*(3.32,222) = 20.3, *p* < 0.001, $\eta_{p}^{2}$ = 0.233] (*p* < 0.05 for all comparisons). There were no significant differences in motor costs between the Clock Task, 2-Back, SST, and Stroop (**Figure 3** left side). Motor costs for the COD- pathway in the number + letters condition were significantly higher than in the numbers condition [*F*(1,65) = 79.9, *p* < 0.001, $\eta_{p}^{2}$ = 0.551] (**Figure 3** right side). *Post hoc* comparisons showed significantly lower motor dual task costs on all tasks on the straight pathway, but not the COD pathway for adolescents compared to children (*p* < 0.05 for all comparisons).

There were also significant differences in the cognitive DTEs across tasks on the straight pathway [*F*(1.94,69.9) = 4.12, *p* = 0.021, $\eta_{p}^{2}$ = 0.103] as well as on the COD pathway [*F*(1,65) = 19.8, *p* < 0.001, $\eta_{p}^{2}$ = 0.245]. The cognitive cost of DT walking was greatest in the TWT-2 condition compared to all other conditions (*p* < 0.01 for all comparisons), whereas the cognitive cost was lowest in the motor task. *Post hoc* comparisons indicated significant better performances in the Clock Task, the Auditory 2-Back Task, and the Auditory Stroop Task for children, but significantly poorer performance for adolescents in the Auditory 2-Back Task, and the Auditory Stroop Task.

**FIGURE 4:**
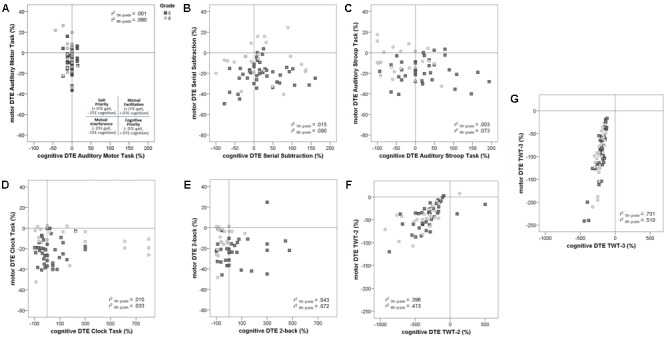
Profiles of cognitive-motor interference across motor and cognitive tasks. DTE refers to dual task effect. Positive values for dual-task effects (DTE) indicate that performance improved in dual-task condition relative to single-task performance; negative values for DTE indicate that performance deteriorated in dual-task condition relative to single-task performance (Plummer and Eskes, 2015). **(A)** Auditory Motor Task; **(B)** Serial Subtraction Task; **(C)** Auditory Stroop Task; **(D)** Clock Task; **(E)** 2-back; **(F)** Trail-Walking-Test – numbers; **(G)** Trail-Walking-Test – numbers and letters.

Individual comparisons of ST and DT conditions showed clear evidence of mutual interference, where motor and cognitive performance declined both under DT conditions, or prioritizing cognitive performance, such that gait speed decreased but cognitive performance improved under DT conditions (see **Figures 4A–G**).

## Discussion

To our knowledge, this is the first study to compare the effects of different types of motor (i.e., straight vs. COD pathway) and cognitive tasks (i.e., non-executive distractor tasks vs. higher-level executive function tasks) on DT performance in children and adolescents. As expected, the main findings indicate that walking on a COD pathway is more difficult than walking on a straight pathway. Cognitive performance differed significantly in the different types of tasks, reflected in better performance for the Auditory Motor Task and the Serial Subtraction Task, followed by the Auditory Stroop Task, the 2-back Task, and the Clock Task (CCR scores), regardless of the ST vs. DT condition. Furthermore, our results show that the Auditory Motor Task was the least demanding task; higher-level executive function tasks were more demanding than non-executive distractor tasks, as reflected in non-significant differences in gait speed for these tasks on a straight pathway and the COD pathway. The calculation of DTEs revealed that motor DTEs were lowest for the Auditory Motor Task and highest for the Trail-Walking-Test in the numbers + letters condition. In contrast, there were cognitive benefits for the higher-order cognitive tasks on the straight pathways, but cognitive costs for both DT conditions on the COD pathway.

### Effect of Different Motor Tasks on Cognitive-Motor Interference

The comparison of different walking tasks and their demands for dual-task walking attracted only little attention so far (Beurskens and Bock, 2013). Only few studies have examined the influence of different physical environments while dual-tasking, either using different terrains or obstacles (Schrager et al., 2008; Beurskens and Bock, 2013; Simoni et al., 2013; Lin and Lin, 2016). However, coping with everyday tasks does not just require walking on straight stretches. The treadmill or straight-walking DTs commonly used in clinical trials and in the DT research literature seem to be too simple in their demand for motor control (due to constant walking speeds and unexpected perturbations) to produce significant interferences (Schott, 2015).

While straightforward walking we observed higher gait speeds in children compared to adolescents. Only when cornering in the COD walking condition, we see an advantage in adolescents, probably due to the aforementioned requirements of the walking tasks. This suggests that cornering in children is not automated to the extent as it is in adolescents. In addition to the assumption of higher motor requirements in children, it is also conceivable that children have greater difficulty to follow the instructions. Subjects should walk at a normal walking pace without racing. During the study it could be observed that some children have tried to walk faster than instructed because they probably interpret it as a competition (lack of inhibitory control, Boelema et al., 2014).

Compared to walking straight ahead, different cognitive functions are addressed when walking on COD pathways. While straightforward walking can be solved by simple information processing, cognitive flexibility and the ability to change tasks explains the speed of cornering (Lowry et al., 2012) and walking with directional changes (Mazaheri et al., 2014). Studies also demonstrate that dual-task-related declines in gait performance are more pronounced during walking tasks requiring greater visual processing and feedforward visual planning, such as obstacle avoidance (Beurskens and Bock, 2013). Precise placement of the feet, especially in difficult environmental conditions to prevent tripping and slipping, is essential (Alexander et al., 2005), primarily visually controlled and requires some level of close attention (Menant et al., 2014). The Trail-Walking-Test follows a COD course characterized by a necessary asymmetry of foot placement and involves steering the body in different directions. Navigation in this task is a rather complex ecological activity involving spatial cognition through body motion using either an egocentric or allocentric frame of reference (Schott et al., 2016). Large-scale spatial tasks can be used to assess either egocentric spatial memory processes, but allocentric memory processes preferentially (Lavenex et al., 2015). Due to their developing executive function (Bullens et al., 2010) children as young as 10 years still exhibit incomplete spatial abilities. They are not fully able to switch between and/or simultaneously use different sources of spatial information and reference frames as it is accomplished, by fully developed adolescents (Belmonti et al., 2013; Broadbent et al., 2014). From this point of view, our results are in agreement with the literature, showing better navigational skills in older than younger children.

Considering the first aim of the study, we hypothesized that compared to straight walking COD walking while dual-tasking participants would exhibit a decrease in performance on the cognitive task. Consistent with our hypothesis, and similar to the results of Schaefer et al. (2010), we see an overall improvement in cognitive performance under DT walking on a straight pathway. In contrast, in the COD walking condition, we observed a considerable decrease in cognitive performance, primarily in the condition with low cognitive load. As the difficulty level of the secondary cognitive task in the COD walking condition increases (numbers + letters), we observe decreased cognitive costs and increased motor costs compared to the lower demanding cognitive task (numbers). Moreover, in the cognitive DT conditions no age-dependent dual-task effects were found on gait velocity regardless of the walking condition.

The beneficial effect on cognitive performance may be explained by general increases in arousal induced by the walking task (e.g., Adam et al., 1997). However, this cannot explain the cognitive decline in the COD condition. Other explanatory theoretical models have been proposed to explain conflicting findings in the locomotion-cognition literature: the Cross-Domain Competition Model (limited attentional and processing capacity in humans; Lacour et al., 2008), the U-shaped non-linear interaction model (cognitive demand of the secondary task can either improve or diminish postural stability; Huxhold et al., 2006), the Task Prioritization Model (subjects always prioritize the gait task over the cognitive activity under specific threatening conditions, known as “posture first” strategy; Shumway-Cook et al., 1997; Yogev-Seligmann et al., 2012), and Constrained Action Hypothesis (external focus facilitates of motor performance due to promoting automatic control of movement; McNevin et al., 2003).

The Cross-Domain Competition Model and the Task Prioritization Model best explain the findings of the present study. However, the “Cross Domain Competition Model” (Lacour et al., 2008) tries to explain that even two tasks that are not identical in their structure can interfere with each other. In particular, the model assumes that balance control and various cognitive tasks (primarily executive functions) compete with each other for identical brain resources, and that EF and the integration of sensory information into locomotion are important issues (Yogev-Seligmann et al., 2008), which might lead to a decline in both tasks simultaneously or in either the motor or the cognitive task performance under DT conditions. In this regard, there are findings demonstrating overlapping neural networks for postural control and visual-spatial tasks (Barra et al., 2006; Sturnieks et al., 2008) and show that visually demanding tasks or mental tracking tasks are particularly sensitive to the production of dual task costs (Al-Yahya et al., 2011; Beurskens and Bock, 2012). In light of the “Cross Domain Competition Model,” the interferences in our study are explained as follows: even simple motor tasks (walking), which are primarily run by subcortical structures are characterized by non-automated processes and take up minimal attentional resources (Koenraadt et al., 2014). However, the required resources in the straight walking condition are low, so there is negligible loss of performance in the motor domain. Under more challenging motor conditions (e.g., avoiding obstacles or COD walking), postural tasks take more cognitive effort. This in turn leads to increased interference in the motor and particular in the cognitive domain. However, when there is a competition for resources, subjects might exhibit an unconscious strategy to prioritize the motor or cognitive task altering the overall motor or cognitive performance.

The “Integrated Model of Task Prioritization” (Yogev-Seligmann et al., 2012) tries to explain why there are different self-selected strategies to handle DT situations and why resource allocation or prioritization varies. The interplay between postural reserve and hazard estimation is crucial and determines which strategy of prioritization will be executed. Recent studies have found that during DT walking healthy young individuals tend to allocate most of their attention to the secondary task unless they perceive high demands from the walking task or are experts in the secondary cognitive task (Plummer and Eskes, 2015). In the current study many younger children prioritized the cognitive task during the walking task with low demands, but not during the motor task with high demands. Children with high postural reserve and hazard estimation are able to prioritize the cognitive task for an extended period without any adverse effects on gait. Thus, unlike older people, we see a “posture-second” strategy during low demanding walking conditions especially with a combination of higher-level EF tasks. When the environment becomes more complex and walking is challenged, the focus of attention shifts toward the motor task to maintain gait stability. These results are consistent with Boonyong et al. (2012) who demonstrated that children (5–6 years, and 7–16 years), apply a more careful strategy with reduced gait speed and step length during obstacle crossing while dual tasking. Schaefer et al. (2008) suggest that children invest more resources into the balance task to avoid putting their balance at risk when overall attentional demand increase. Even if a performance deterioration in the motor task and the associated possible consequences such as falls, do not have the same ecological relevance in children as in the elderly (Li et al., 2005), this tendency shows that also young children are able to exhibit healthy risk judgment (Yogev-Seligmann et al., 2012).

### Effect of Different Cognitive Tasks on Cognitive-Motor Interference

As already highlighted before, studies in the field of motor-cognitive DTs exhibit an enormous variation in the use of cognitive tasks. However, different components of EF have shown to develop at different rates throughout childhood (Boelema et al., 2014). Therefore, the results for the relationship of motor and cognitive performance rely highly on the selected cognitive task. Similar to the study results of Walshe et al. (2015), we see that the task with low task complexity like the Auditory Motor Task appears to be easier to accomplish, and tasks that require higher-level EF like the Auditory Stroop Task appear to be significantly more difficult. Also in our study the Clock Task seems to have the strongest effect and seems to be the most demanding task. Walshe et al. (2015) suggest that this could be due to a doubling of executive components (both working memory and visuo-spatial imagining of the clock face) and could thus further tax the EF processing capacities.

With a closer look on the absolute times in the DT conditions, we can observe differences between the different cognitive tasks in the straight pathway walking condition. In both age groups the simple Auditory Motor Task did not increase the walking performance and thus stands in contradiction to the “U-Shaped Non-linear Interaction Model” (Lacour et al., 2008) and contrary to the hypothesis of constrained action (Wulf et al., 2001). This model states that an easy cognitive task can lead to an external focus of attention. An external focus on a highly automatic skill – such as walking – can improve the automatic control processes and enables a self-organized postural control system to facilitate walking. An internal focus of attention, on the other hand, which we could expect in the ST, disrupts the automatic control processes and could impair walking performance. However, as we do not see any improvement with the Auditory Motor Task, our results contradict to this model. In regard of the DT effects, this study demonstrates that, when completing a secondary task that taxes higher-level cognition, DT changes in gait are attributable to more than low-level motor response processes. These effects specifically show the direct competition for higher-level EF resources while walking and are in agreement with previous studies supporting the EF-motor link in relation to gait (Mirelman et al., 2012). Unlike Walshe et al. (2015), however, in children or adolescents we see no clear difference in dual task costs between these different higher-level EF tasks in the walking straight condition. Indeed, in the more challenging COD walking condition we observe that gait speed decreases with increasing task difficulty. It seems that the most cognitive challenging dual-task paradigms for children are those in which visual scanning of the external environment is required (Matthis et al., 2017). In this respect, in addition to visual scanning also cognitive processing speed, linguistic, executive and attention components are recorded with the TWT (Schott, 2015). However, since this is a mobile version of the TMT (Reitan, 1955) and the TMT primarily allows a statement about cognitive flexibility (Crowe, 1998) and is probably the most widely used tool for assessing the ability to change tasks (Arbuthnott and Frank, 2000), there seems to be a strong correlation between the construct of cognitive flexibility and complex locomotion tasks. Ble et al. (2005), Hirota et al. (2008) as well as Killane et al. (2014) were able show that individuals with poor performance in cognitive flexibility have difficulty controlling their gait and adapting it to increased motor demands. The aforementioned connection between the construct of cognitive flexibility and locomotion manifests itself inter alia in the fact that the prefrontal cortex is active both, in the processing of the TMT and in locomotor tasks (La Fougere et al., 2010; Lee et al., 2014). This indicates the sharing and common use of the prefrontal cortex and associated neuronal areas when performing the TWT and explains why we observe the most interference in this study.

### Study Limitations and Future Directions

The results of this study suggest that cognitive and motor DT gait evaluation may be incorporated into the evaluation of executive functions. However, some limitations to this study should be noted. This study’s limitations mainly encompass the cross-sectional design. Due to this design, a causal relationship between maturation and the observed findings regarding walking measured under single and dual-task conditions cannot be drawn. Longitudinal studies are needed to detect the factors, such as physical and cognitive improvements, that may more directly contribute to attentional demands exceeding total capacity in dual-task performance across childhood. In addition, measures such as the Tanner stage, hormonal assays, or a combination of techniques should be used, when examining DT performance in participants in the puberty stage (Dorn et al., 2006).

Furthermore, the quantification of children’s gait performance was characterized by only a single quantitative parameter (duration), but not qualitative parameters. As different variables (duration and distance) are used, the comparison of conditions is only possible to a limited extent. When interpreting the results and differences in straight walking or COD walking this must be taken into account. Although duration is a typical measurement in DT literature, other studies have shown that spatiotemporal patterns are differentially related to dual-task performance (Kraan et al., 2017). Therefore, future studies should include metrics that quantify parameters such as step- and stride length, double support time, head and body movements. However, using a more complex walking route rather than walking on a straight pathway increased the ecological validity of our walking task. The aim is to generate as realistic as possible situations through the respective test procedure. Moreover, no instructions were given to subjects regarding task prioritization: however, young children are able to exhibit healthy risk judgment (Yogev-Seligmann et al., 2012). They allocate most of their attention to the motor task when they perceive high demands from the walking task. It would be interesting to see if this is true even for older adults. This is crucial in order to make a statement regarding resource allocation strategies in the elderly and to assess their risk of falling (Schott and Klotzbier, unpublished). It also seems important to mention that it cannot be said with absolute certainty that the difficulty of the motor tasks with the frequent changes of direction (COD) in the TWT is responsible for the increased costs. It could well be that only the visual claim is decisive. We did not have any visually demanding requirements in any straightforward dual task condition. Future studies should consider different levels of difficulty in locomotion tasks with visually challenging additional cognitive tasks to better understand the relative demands for attention.

Another limitation was that conditions were not counterbalanced for ST and DT and therefore, results can only be interpreted in the context of STs occurring first and DTs occurring after all STs had been performed. Despite this, children and adolescents were faster in the ST conditions, and their performance deteriorated with increased task difficulty in the DT conditions. Thus, if anything, counterbalancing may have increased the magnitude of the observed differences.

Last but not least a better understanding of the neural mechanisms of DT effects as well as the involvement of EF in DT performance might help to use DT training in clinical populations (Leone et al., 2017).

## Conclusion

Our findings demonstrate that when completing a secondary task that involve higher-level cognition, DT changes in walking (straight as well as COD-pathways) are more pronounced than low-level divided attention or motor response processes. These results specifically show the direct competition for higher-level EF resources important for walking, and are in agreement with previous studies supporting the EF-motor link in relation to gait in children as well as older adults (Walshe et al., 2015; Saxena et al., 2017). This observation is particularly notable in complex locomotion tasks as our study shows and is in line with the idea that younger children may not have adequate cognitive resources. Walshe et al. (2015, p. 9) claim that an “underlying executive control system operates as an orchestrating body, allocating resources to and integrating information from the sensory inputs necessary for complex real-world walking.” In future studies, consideration should increasingly be given to more ecologically valid locomotion tasks in order to investigate motor-cognitive interferences in children.

## Author Contributions

NS and TK contributed to writing, data analysis, and results. TK contributed to data collection and wrote the computer program for the cognitive tasks.

## Conflict of Interest Statement

The authors declare that the research was conducted in the absence of any commercial or financial relationships that could be construed as a potential conflict of interest.

## Footnotes

1 https://nccd.cdc.gov/dnpabmi/Calculator.aspx?CalculatorType=Metric
