# Supplementary material for: Profiles of Cognitive-Motor Interference During Walking in Children: Does the Motor or the Cognitive Task Matter?
Source: Front Psychol. 2018 Jun 13;9:947. doi: 10.3389/fpsyg.2018.00947 (PMC6008773; doi:10.3389/fpsyg.2018.00947)
Supplement: Supplementary file 1 [file Table_1.DOCX]

**Supplementary material**

***Table S1*** Correlation coefficients resulting from the univariate correlation analysis for demographics, physical activity, subjective motor performance, and motor and cognitive performance

|  |  | **Age** | **Sex** | **BMI** | **Exercise** | **MABC AB** | **MABC C** | **VO_2_max** |
| --- | --- | --- | --- | --- | --- | --- | --- | --- |
| **ST gait speed  straight pathway** (m/s) |  | **-.341**** | .041 | -.140 | -.061 | .033 | **.277*** | .114 |
| **Information processing speed  (Auditory Motor Task)** | DT gait speed CCR ST CCR DT DTE motor DTE cognitive | -.090 .102 -.152 **.340*** -.226 | .054 -.091 -.108 .032 -.030 | -.120 .112 -.052 .070 -.087 | .073 .173 -.120 .203 -.125 | -.075 -.172 .296 -.215 **.308*** | .229 **-.253*** .152 -.115 .147 | .128 .081 -.072 -.061 -.121 |
| **Verbal WM  (Serial Subtraction Task)** | DT gait speed CCR ST CCR DT DTE motor DTE cognitive | -.012 .029 -.025 **.362**** -.070 | .139 **-.273* -.308*** .088 -.126 | -.246 .160 -.030 -.054 -.076 | .078 **.272*** .062 .175 -.229 | -.109 -.100 -.056 **-.239*** .018 | .110 -.078 -.063 -.214 .024 | .153 **.391** .390**** .046 -.040 |
| **Auditory WM  (Auditory 2-Back Task)** | DT gait speed CCR ST CCR DT DTE motor DTE cognitive | .146 **.333**** .008 **.583** -.386**** | .093 .026 -.064 .070 -.044 | .148 .099 .048 **.310*** -.147 | .246 .187 .013 **.354**** -.157 | -.121 -.009 -.023 -.203 .132 | .032 -.007 .085 **-.257*** -.051 | .076 .138 .028 .001 -.237 |
| **Visuo-spatial WM  (Clock Task)** | DT gait speed CCR ST CCR DT DTE motor DTE cognitive | .156 -.430** **-.349** .571**** .094 | .226 -.237 .123 .188 **.308*** | .086 -.168 -.157 **.297*** .025 | .175 -.026 .008 **.278*** .088 | -.035 .085 .077 -.100 .014 | .093 .024 .144 **-.283*** .113 | .014 .081 -.226 -.075 -.211 |
| **Response inhibition  (Auditory Stroop Task)** | DT gait speed CCR ST CCR DT DTE motor DTE cognitive | .245 **.249*** -.020 **.514** -.433**** | .034 .158 .105 .018 .021 | .140 .146 .118 .234 -.093 | .188 .209 -.137 **.259* -.319*** | -.039 -.165 .048 -.166 .169 | .218 -.035 .035 .-106 .175 | .114 .045 -.168 -.021 **-.340**** |
| **ST gait speed  COD-pathway** (m/s) |  | **.245*** | .034 | .140 | .188 | -.039 | .218 | .114 |
| **Information processing speed (TMT A** [cm/s] **/ TWT-2** [m/s]**)** | DT tracing speed DT gait speed DTE motor DTE cognitive | **.267*** .078 -.156 -.165 | .195 .024 .057 -.236 | .218 -.003 -.120 -.144 | .126 .058 -.177 -.014 | -.255 .122 152 .173 | -.217 .196 .046 .193 | -.165 .004 -.077 .177 |
| **Cognitive flexibility (TMT B** [cm/s] **/ TWT-3** [m/s]**)** | DT tracing speed DT gait speed DTE motor DTE cognitive | **.344**** .077 -.067 -.184 | -.004 .061 -.026 .087 | -.013 .129 .052 -.005 | **.280*** -.031 -.081 -.157 | -.135 .114 .213 .188 | .116 .189 .020 .032 | **.244*** -.017 -.009 -.121 |

Notes: BMI Body Mass Index; MABC Movement Assessment Battery; ST Single Task; DT Dual Task; DTE Dual Task Effects; CCR correct cognitive response; * indicates *p* < .05; ** indicates *p* < .05; significant results in bold

***Table S2*** Results from the multiple regression analysis with stepwise regression

|  |  |  | **Standardized beta** | **t** | **p** | **Partial R^2^** | **R^2^** | **Significance of model** |
| --- | --- | --- | --- | --- | --- | --- | --- | --- |
| **ST gait speed  straight pathway** (m/s) |  | age | -.320 | -2.59 | .012 | .087 | .087 | .012 |
| **Information processing speed  (Auditory Motor Task)** | DT gait speed CCR ST CCR DT DTE motor DTE cognitive | - - - age MABC AB | - - - .390 .313 | - - - 3.26 2.40 | - - - .002 .020 | - - - .152 .098 | - - - .138 .081 | - - - .002 .020 |
| **Verbal WM  (Serial Subtraction Task)** | DT gait speed CCR ST  CCR DT DTE motor  DTE cognitive | - VO_2_max BMI VO_2_max age BMI - | - .438 .244 .357 .428 -.267 - | - 3.70 2.06 2.91 3.34 -2.08 - | - .001 .044 .005 .001 .042 - | - .155 .058 .128 .144  - | - .186  .113 .110 .062 - | - .001  .005 .004  - |
| **Auditory WM  (Auditory 2-Back Task)** | DT gait speed CCR ST CCR DT DTE motor DTE cognitive | - age - age age | - .380 - .506 -.405 | - 3.15 - 4.51 -2.91 | - .003 - <.001 .006 | - .144 - .256 .164 | - .130 - .244 .145 | - .003 - <.001 .006 |
| **Visuo-spatial WM  (Clock Task)** | DT gait speed CCR ST CCR DT DTE motor DTE cognitive | - age age age age | - -.427 -.326 .518 .378 | - -3.56 -2.63 4.65 2.97 | - .001 .011 <.001 .004 | - .182 .106 .268 .143 | - .168 .091 .256 .127 | - .001 .011 <.001 .004 |
| **Response inhibition  (Auditory Stroop Task)** | DT gait speed CCR ST CCR DT DTE motor DTE cognitive | - age Exercise age age MABC AB | - .327 -.258 .464 -.362 .300 | - 2.66 -2.03 4.03 -2.90 2.41 | - .010 .047 <.001 .005 .020 | - .107 .066 .216 .198 .083 | .- .092 .050 .202 .253 | - .010 .047 <.001 <.001 |
| **ST gait speed  COD-pathway** (m/s) |  | Exercise MABC C | .309 .252 | 2.52 2.06 | .015 .044 | .078 .063 | .111 | .012 |
| **Information processing speed (TMT A** [cm/s] **/ TWT-2** [m/s]**)** | DT tracing speed  DT gait speed DTE motor DTE cognitive | MABC AB VO_2_max - - - | -.336 -.276 - - - | -2.65 -2.17 - - - | .010 .034 - - - | .067 .070 - - - | .107  - - - | .014  - - - |
| **Cognitive flexibility (TMT B** [cm/s] **/ TWT-3** [m/s]**)** | DT tracing speed DT gait speed DTE motor DTE cognitive | age - - - | .291 - - - | 2.33 - - - | .023 - - - | .085 - - - | .069 - - - | .023 - - - |

Notes: BMI Body Mass Index; MABC Movement Assessment Battery; ST Single Task; DT Dual Task; DTE Dual Task Effects; CCR correct cognitive response; * indicates *p* < .05; ** indicates *p* < .05

***Table S3.*** Performance outcome measures in ST and DT conditions by grade (means ± standard deviation)

|  |  | **5^th^ grade** | **8^th^ grade** | **statistical analysis** |
| --- | --- | --- | --- | --- |
|  |  | **(*n*=42)** | **(*n*=27)** |  |
| **ST gait speed  straight pathway** (m/s) |  | 1.90 ± 0.29 | 1.73 ± 0.29 | *t*(67) = 2.47, *p* = .016, d = -0.59 |
| **Information processing speed  (Auditory Motor Task)** | DT gait speed CCR ST CCR DT DTE motor DTE cognitive | 1.73 ± 0.32 0.25 ± 0.03 0.25 ± 0.03 -8.97 ± 10.4 -2.09 ± 9.31 | 1.69 ± 0.27 0.26 ± 0.01 0.25 ± 0.03 -1.33 ± 13.8 -5.98 ± 11.5 | *t*(67) = 0.60, *p* = .552, d = -0.13 *t*(54) = -1.80, *p* = .078, d = 0.38 *t*(65) = 0.73, *p* = .469, d = -0.18 *t*(67) = -2.62, *p* = .011, d = 0.65 *t*(59) = 1.46, *p* = .151, d = -0.38 |
| **Verbal WM  (Serial Subtraction Task)** | DT gait speed CCR ST CCR DT DTE motor DTE cognitive | 1.49 ± 0.27 0.20 ± 0.09 0.23 ± 0.12 -21.3 ± 11.5 19.7 ± 53.4 | 1.50 ± 0.32 0.22 ± 0.15 0.24 ± 0.14 -11.7 ± 17.9 18.9 ± 66.9 | *t*(67) = -0.19, *p* = .847, d = 0.03 *t*(37) = -0.72, *p* = .479, d = 0.20 *t*(65) = -0.36, *p* = .719, d = 0.09 *t*(67) = -2.47, *p* = .018, d = 0.67 *t*(62) = 0.57, *p* = .955, d = -0.01 |
| **Auditory WM  (Auditory 2-Back Task)** | DT gait speed CCR ST CCR DT DTE motor DTE cognitive | 1.47 ± 0.35 0.05 ± 0.07 0.06 ± 0.06 -22.6 ± 14.6 69.6 ± 160 | 1.55 ± 0.28 0.12 ± 0.08 0.07 ± 0.06 -9.32 ± 12.1 -48.0 ± 54.2 | *t*(67) = -1.02, *p* = .309, d = 0.25 *t*(67) = -3.44, *p* = .001, d = 0.85 *t*(66) = -0.42, *p* = .673, d = 0.11 *t*(67) = -3.94, *p* < .001, d = 0.97 *t*(39) = 3.80, *p* < .001, d = -0.92 |
| **Visuo-spatial WM  (Clock Task)** | DT gait speed CCR ST CCR DT DTE motor DTE cognitive | 1.46 ± 0.27 0.05 ± 0.03 0.05 ± 0.03 -23.1 ± 11.6 21.3 ± 96.2 | 1.55 ± 0.30 0.02 ± 0.02 0.02 ± 0.02 -9.74 ± 13.2 189 ± 316 | *t*(67) = -1.31, *p* = .196, d = 0.32 *t*(65) = 4.31, *p* < .001, d = -0.97 *t*(66) = 3.63, *p* = .001, d = -0.83 *t*(67) = -4.42, *p* < .001, d = 1.09 *t*(24) = -2.48, *p* = .021, d = 0.82 |
| **Response inhibition  (Auditory Stroop Task)** | DT gait speed CCR ST CCR DT DTE motor DTE cognitive | 1.54 ± 0.30 0.09 ± 0.05 0.10 ± 0.04 -18.9 ± 11.6 22.4 ± 64.8 | 1.56 ± 0.26 0.11 ± 0.06 0.10 ± 0.07 -8.69 ± 12.6 -34.9 ± 53.3 | *t*(67) = -0.33, *p* = .742, d = 0.07 *t*(67) = -1.59, *p* = .116, d = 0.39 *t*(38) = 0.37, *p* = .716, d = -0.10 *t*(67) = -3.45, *p* = .001, d = -0.59 *t*(58) = .55, *p* < .001, d = -0.94 |
| **ST gait speed COD-pathway** (m/s) |  | 1.46 ± 0.22 | 1.58± 0.19 | *t*(67) = -2.32, *p* = .023, d = 0.58 |
| **Information processing speed (TMT A** [cm/s] **/ TWT-2** [m/s]**)** | DT tracing speed DT gait speed DTE motor DTE cognitive | 6.14 ± 1.54 1.05 ± 0.21 -44.2 ± 29.3 -311 ± 227 | 7.06 ± 1.32 1.09 ± 0.18 -48.1 ± 27.0 -374 ± 232 | *t*(67) = -2.56, *p* = .013, d = 0.63 *t*(67) = -1.02, *p* = .312, d = 0.20 *t*(67) = 0.55, *p* = .581, d = -0.14 *t*(61) = 1.07, *p* = .293, d = -0.28 |
| **Cognitive flexibility (TMT B** [cm/s] **/ TWT-3** [m/s]**)** | DT tracing speed DT gait speed DTE motor DTE cognitive | 3.40 ± 0.87 0.78 ± 0.23 -92.0 ± 54.1 -215 ± 88.8 | 4.00 ± 0.82 0.83 ± 0.17 -98.8 ± 47.5 -217 ± 49.9 | *t*(67) = -2.86, *p* = .006, d = 0.71 *t*(67) = -0.81, *p* = .422, d = 0.24 *t*(65) = 0.53, *p* = .597, d = -0.13 *t*(67) = 0.10, *p* = .924, d = -0.03 |
